# Clinical utility of complex assessment with evoked potentials in acute lymphoblastic leukemia survivors: comparison of various treatment protocols

**DOI:** 10.1186/s12885-021-07873-x

**Published:** 2021-02-10

**Authors:** Slawomir Kroczka, Konrad Stepien, Izabela Witek-Motyl, Kinga Kwiecinska, Eryk Kapusta, Agnieszka Biedron, Pawel Skorek, Szymon Skoczen

**Affiliations:** 1 Chair of Child and Adolescent Neurology, Jagiellonian University Medical College, Krakow, Poland; 2 Department of Childhood Neurology, University Children’s Hospital, Krakow, Poland; 3 Department of Oncology and Hematology, University Children’s Hospital, 265 Wielicka St., 30-663 Krakow, Poland; 4 Department of Pediatric Oncology and Hematology, Institute of Pediatrics, Jagiellonian University Medical College, Krakow, Poland

**Keywords:** Acute lymphoblastic leukemia, Children, Survivors, Evoked potentials, Radiotherapy

## Abstract

**Background:**

One of the greatest success of pediatric hematology is a prominent improvement of survival in acute lymphoblastic leukemia (ALL*).* Therefore*,* special attention needs to be paid to long-term side effects of the treatment such as neurotoxicity. One of the few diagnostic methods that allow an objective assessment of sensory systems are evoked potentials (EP).

**Methods:**

The analyzed group consisted of 167 ALL long-term survivors, aged 4.9–28.4 years, without auditory, visual and sensory deviations. Patients were treated with New York (NY, *n* = 35), previous modified Berlin-Frankfurt-Münster (pBFM, *n* = 47) and BFM95 (*n* = 85) protocols. In order to assess the impact of radiotherapy on recorded EP, a joint analysis of NY and pBFM groups was performed. The control group consisted of 35 patients, aged 6–17 years. The analyzed patients underwent a complex assessment with visual EP (VEP), somatosensory EP (SEP) and brainstem auditory EP (BAEP) in accordance with current standards.

**Results:**

ALL treatment contributed to the shortening of wave I latency (1.59 vs 1.90, *P* = 0.003) and prolongation of I-III (2.23 vs 2.04, *P* = 0.004) and I-V (4.57 vs 4.24, *P* = 0.002) interwave latencies of BAEP. A significant effect was also noticed in P100 (106.32 vs 101.57, *P* < 0.001) and N135 (151.42 vs 138.22, *P* < 0.001) latencies of VEP and N18 amplitude (3.24 vs 4.70, *P* = 0.007) and P25 latency (21.32 vs 23.39, *P <* 0.001) of SEP. The distribution of abnormalities between protocols was similar in BAEP (NY - 68.6%, pBFM - 61.7%, BFM95–69.4%, *P* = 0.650), VEP (NY - 68.6%, pBFM - 42.5%, BFM95–58.3%, *P* = 0.053) and significantly different for SEP (NY - 62.9%, pBFM - 36.2%, BFM95–53.0%, *P* = 0.045). The harmful effect of radiotherapy was most clearly marked in numerous disturbances of SEP parameters.

**Conclusions:**

The presented analysis indicates a high frequency of subclinical abnormalities in EP regardless of the analyzed protocol. To our knowledge current study is the largest and one of the most complex research examining the role of EP in ALL patients. The obtained results indicate the possibility of using a single, objective and non-invasive measurement of EP in ALL survivors in order to stratify the risk of developing sensory abnormalities in adulthood.

## Background

One of the greatest success of pediatric hematology is a prominent improvement of survival in acute lymphoblastic leukemia (ALL), which is precisely related with introduction of new therapeutic regimens [1]. Nowadays ALL therapeutic protocols consisted mainly of chemotherapy and exceptionally radiotherapy which is associated with potential severe adverse effects. Those factors may lead also to significant changes in the nervous system. According to the fact that ALL is the most common type of cancer in a pediatric population, long-term results of the treatment represent an important social problem [2]. Improvement of survival rates encourage to focus on long-term side effects of the treatment and associated with them quality of life.

Child development is a complex process with an important role of sensory systems. As established earlier, the development of individual senses begins in the early fetal life and is stimulated by various endo- and exogenous stimuli [3]. Adequate sensual growth is crucial for the child’s further development. To date, a number of non-inherited, postnatal factors have been associated with an adverse effect on the hearing, vision and sensory perception development [3]. One of them is undoubtedly intensive anticancer treatment in patients with ALL. Currently used anticancer regimens impair various functional cognitive processes in ALL survivors [4]. Significant changes in the central nervous system such as smaller volumes of neocortical and subcortical gray matter have been demonstrated using magnetic resonance methods [5]. However, clinically observed hearing, vision or sensory impairment in ALL survivors that may be associated with anticancer treatment is rare [6].

One of the few diagnostic methods that allow an objective assessment of individual senses, as well as the whole integrated development of sensory organization are evoked potentials (EP). EP are defined as specific responses to selected, sensory stimuli and may be non-invasively recorded. Multiple types of exogenous, sensory modality can be measured with different modifications of this technique: visual evoked potentials (VEP), somatosensory evoked potentials (SEP), brainstem auditory evoked potentials (BAEP). Peak amplitudes and latencies of particular waves are analyzed in detail [7]. In our previous study we reported that abnormal BAEP can be registered in 22.4% of children treated for ALL [8]. As previously shown by Kaleita et al. [9], Russo et al. [10], Muchi et al. [11] and Korinthenberg et al. [12] abnormalities in EP analysis can be found in a significant proportion of ALL patients. However, there were also some reports in the literature questioning the clinical usefulness of EP in this group of patients [13, 14].

The aim of our study was to conduct and verify the clinical utility of complex neurophysiologic evaluation using different types of EP in group of childhood ALL survivors treated with various protocols.

## Methods

A group of 167, 103 males, aged 4.9–28.4 years who have completed ALL therapy was enrolled to the study. The mean patients age at diagnosis was 5.3 ± 3.5 and 13.9 ± 5.3 at neurophysiological assessment. The time between the end of treatment and the study varied from 0.3 to 20.9 years. Patients at study.The study group was divided into 3 subgroups according to treatment protocols introduced gradually by Polish Leukemia/Lymphoma Study Group. The first group consisted of 35 patients (21 boys, 60%) who received treatment according to modified New York programs (NY). The second group of 47 patients (24 boys, 51.1%) was treated with previously modified BFM protocols (pBFM): BFM 81, 83, 86 and 87 (Table 1). In those two groups optionally, therapeutic central nervous system radiotherapy was conducted. The last group of 85 patients (40 males, 47.1%) underwent treatment with BFM95 protocol without radiotherapy. There were no symptoms of hearing, sight and sensory perceptions disorders in participants. ALL relapse has not been registered in the study population. The central nervous system involvement was confirmed in 7 cases (1 – NY, 5 – pBFM and 1 – BFM95, respectively).

**Table 1:** The most important differences between modified BFM protocols

Protocol/differences	Modified BFM87	Modified BFM95
MTX doses in consolidation	1 g/m^2^	3 g/m^2^
Prophylactic radiotherapy	18Gy	–

Average cumulative dose of vincristine in NY protocols was 60.8 mg/m^2^ and 30 mg/m^2^ in BFM protocols respectively. The radiotherapy dose in pBFM group was 13–36.4 Gy (mean 18.4 Gy), while in the group treated with NY protocols - 18.2-24 Gy (mean 18.3 Gy). It was the whole brain irradiation to the C2 region including retroocular and bases of frontal regions areas. Exclusion criteria from the study were other diagnoses than ALL, patients with primary neurologic diseases and/or inborn genetic defect (Table 2).

**Table 2:** Comparison of clinical and neurological data in irradiated and non-irradiated patients

Parameters	NY *N =* 35	pBFM *N =* 47	BFM95 *N =* 85	Control group *N =* 35
Age at diagnosis, years	6.5 ± 0.5	4 ± 2.7	5 ± 2.6	–
Age at study, years	14 ± 5.6	18.3 ± 4.2	11.4 ± 4.1	11.6 ± 3.6
Median CRT dose, Gy	18.4	18.3	–	–
CNS involvement, N	1	5	1	–
Average cumulative VCR dose, mg/m^2^	60,8	30	30	–
Symptoms of hearing, sight and sensory perceptions disorders	No	No	No	No

Abbreviations: *BFM* Berlin-Frankfurt-Münster protocol, *NY* New York protocol, *CRT* cranial radiotherapy, *VCR* vincristine

The control group consisted of 45 patients (23 males, 51.4%), aged 6–17(mean 11,6 ± 3,6) years. They were patients of Neurology Department inpatient and outpatient, and Outpatient Orthopedic Department of University Children’s Hospital (children with educational difficulties, emotional disorders, children after a single syncope episode) and general healthy volunteers which were neurologically consulted.

The performed studies were as follow: BAEP – 25, VEP - 32, and SEP- 30. No patient in the study and control groups was diagnosed with focal CNS symptoms, in all psychomotor development were in normal range. No one was treated for at least 1 week before neuro-physiological evaluation. They attendant schools according to age.

The evoked potentials evaluations were performed according to IFCN recommendations. The patients were studied in comfortable half-siting position. All were examined in the same room with the use of the same equipment by the same technician. The external conditions were limited with the use of air-conditioner keeping the same temperature and humidity. The obtained data were evaluated by the same physician.

The study protocol was complied with the Declaration of Helsinki and was approved by the Jagiellonian University Medical College Ethics Committee (Consent No. KBET/131/B/207). All parents as well as adolescent and adult patients signed written informed consent before inclusion in the study.

### Auditory evoked potentials methodology

**Fig. 1:**
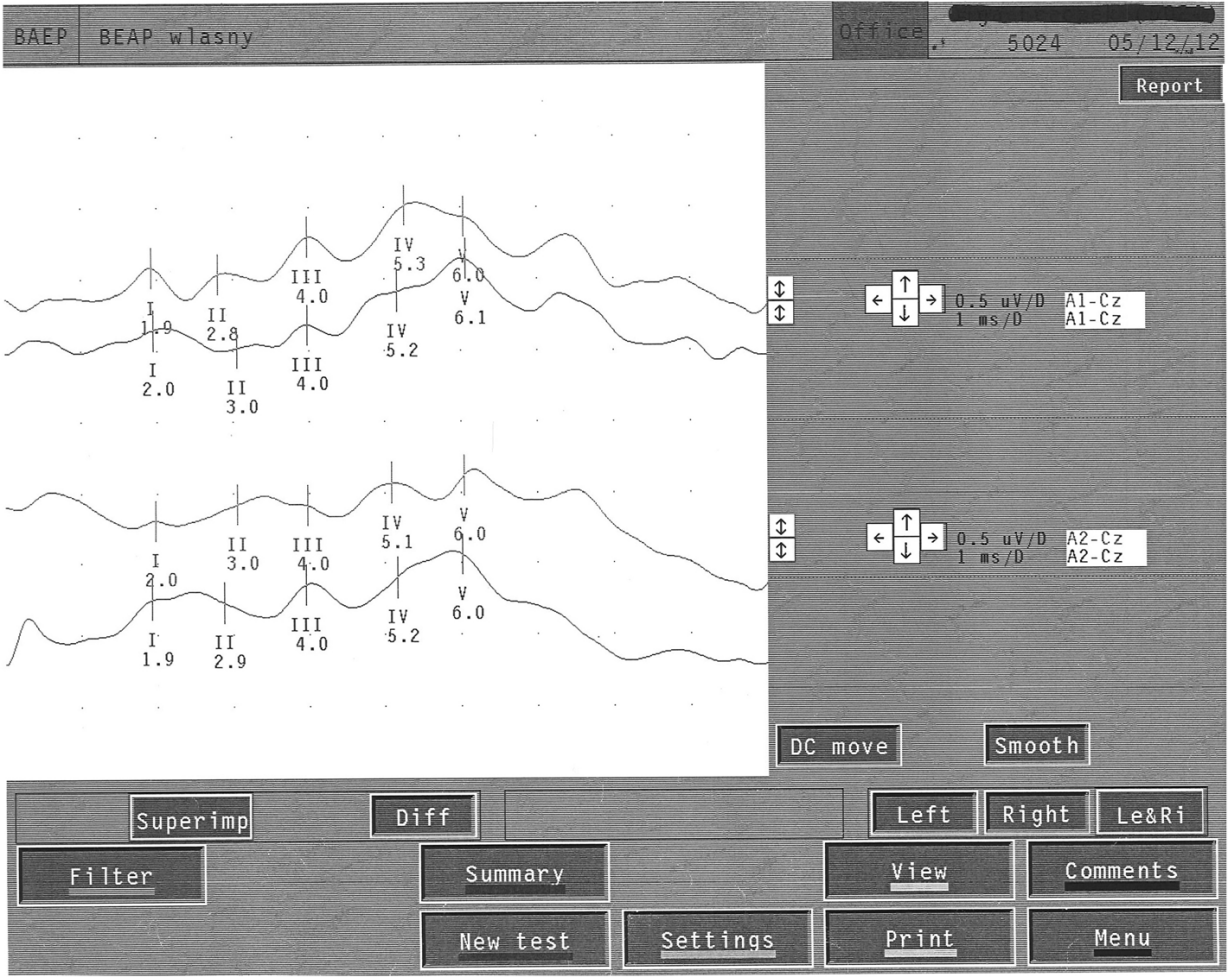
Normal BAEP in 10 y.o. boy

**Fig. 2:**
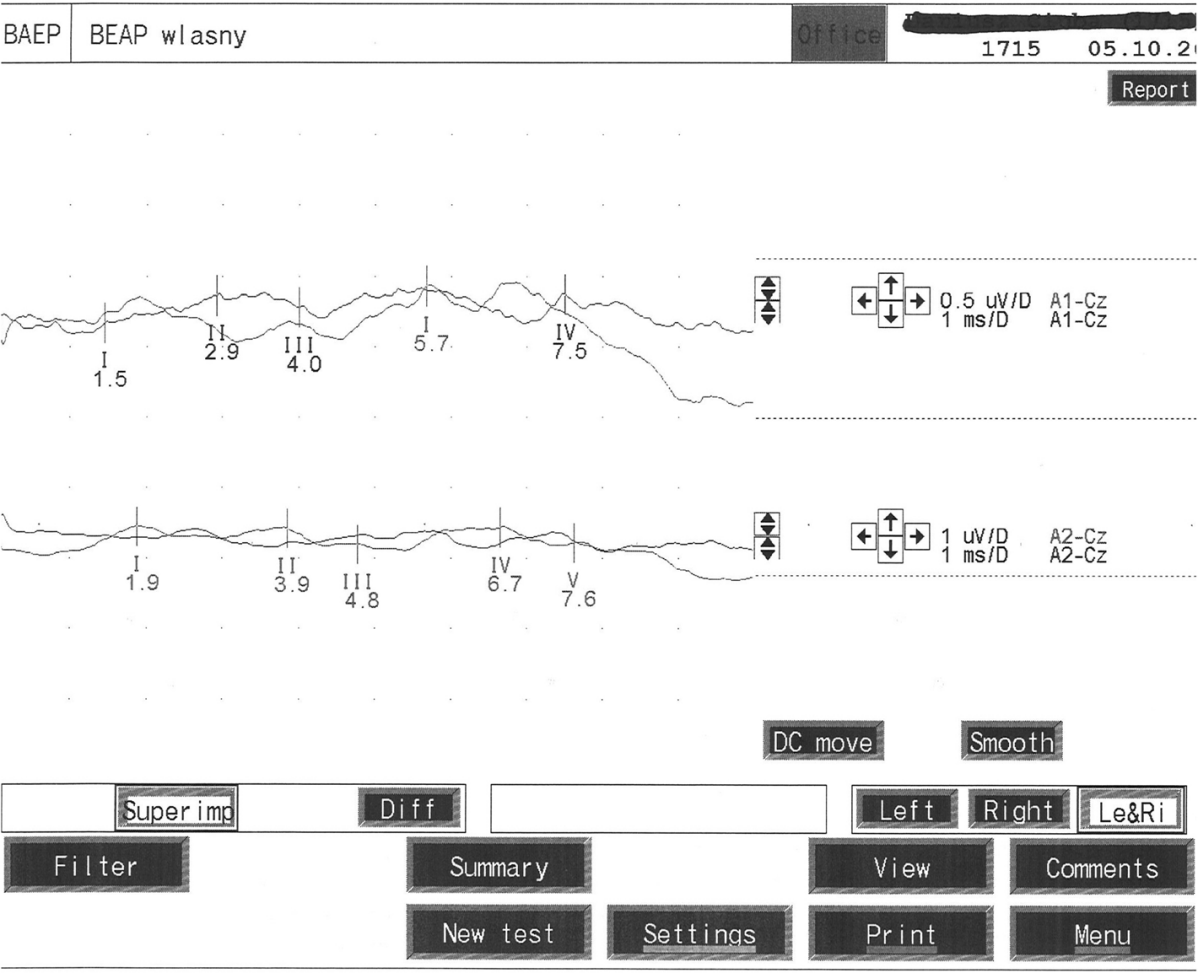
BAEP with elongation of wave V in 10 y.o. boy after ALL treatment

Examination was performed in comfortable semi-sitting position on armchair with back and head support. The stimulation was performed by headphones with use an acoustic impulse. At first, an electrophysiological hearing threshold was estimated. Next, both ear canals were stimulated alternately with repetitive (10 Hz) acoustic stimulus (click) at 70 dB above hearing threshold. During it other ear was masked by murmur at 40 dB lower than acoustic stimulus. BAEP were recorded by using cup electrodes placed according to 10–20 international system. Detected waves were analyzed by comparing impulses from stimulated or not stimulated ear with those from a vertex. Used filter excluded frequencies below 150 Hz and above 300 Hz. Recorded 1000–2000 responses were averaged twice. According to guidelines of International Federation of Clinical Neurophysiology (IFCN) only curves with differences less than 0.1 ms in latencies and less than 10% in amplitudes between waves were taken into consideration. Waves I, III and V, their latencies and also interwave latencies between waves I-V, I-III, III-V were evaluated. Recorded results were compared with normal values based on our own data. Latencies and interwave latencies elongation more than 2 SD were considered as abnormal (Figs. 1 and 2). To evaluate the effect of treatment, comparisons of ALL patients (NY, pBFM, BFM95) were made with the control group. Additionally, to evaluate impact of radiotherapy a group of patients receiving radiotherapy (NY and pBFM) was compared to non-irradiated (BFM95).

### Visual evoked potentials methodology

**Fig. 3:**
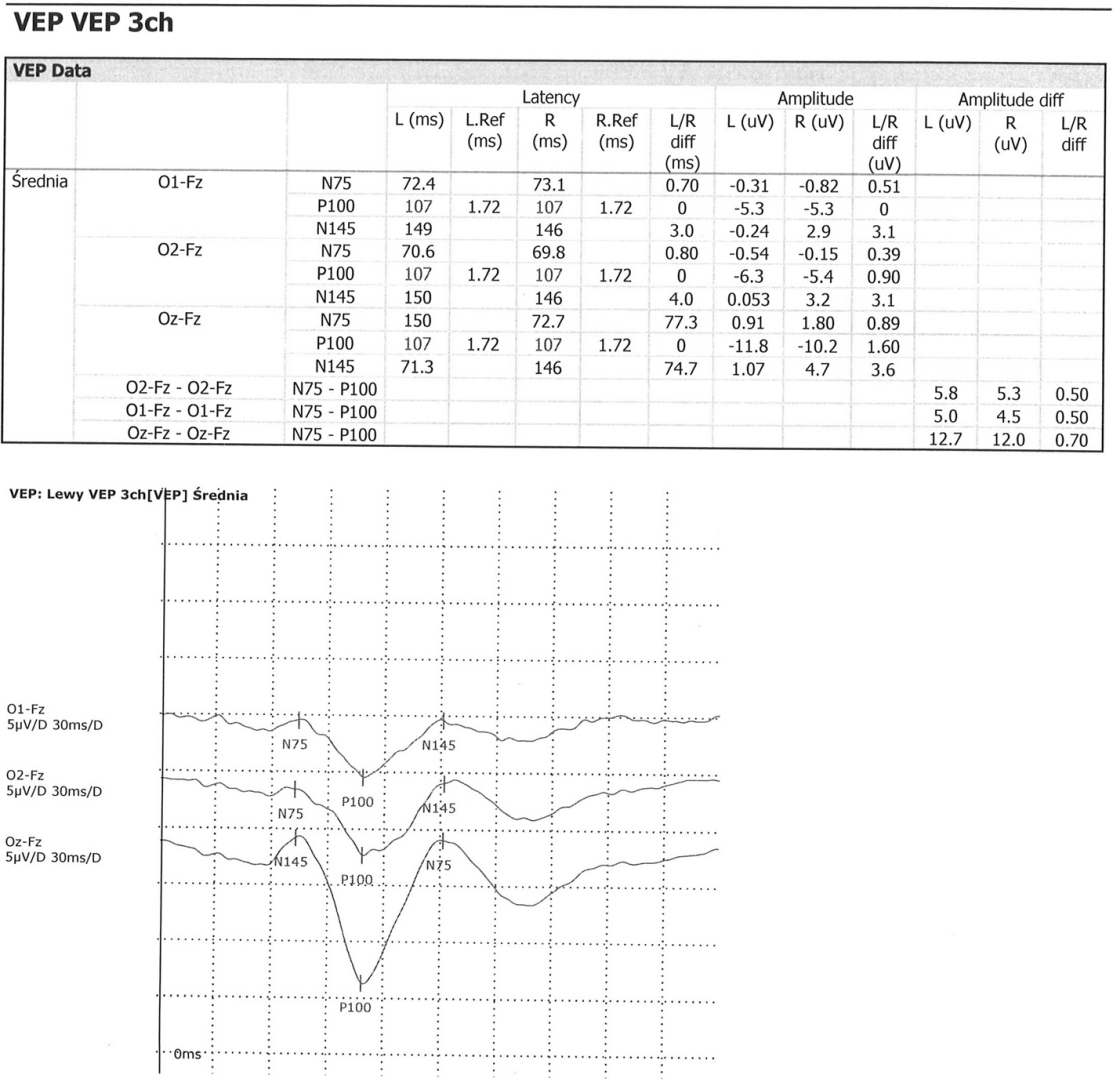
Normal VEP in 7 y.o. girl

**Fig. 4:**
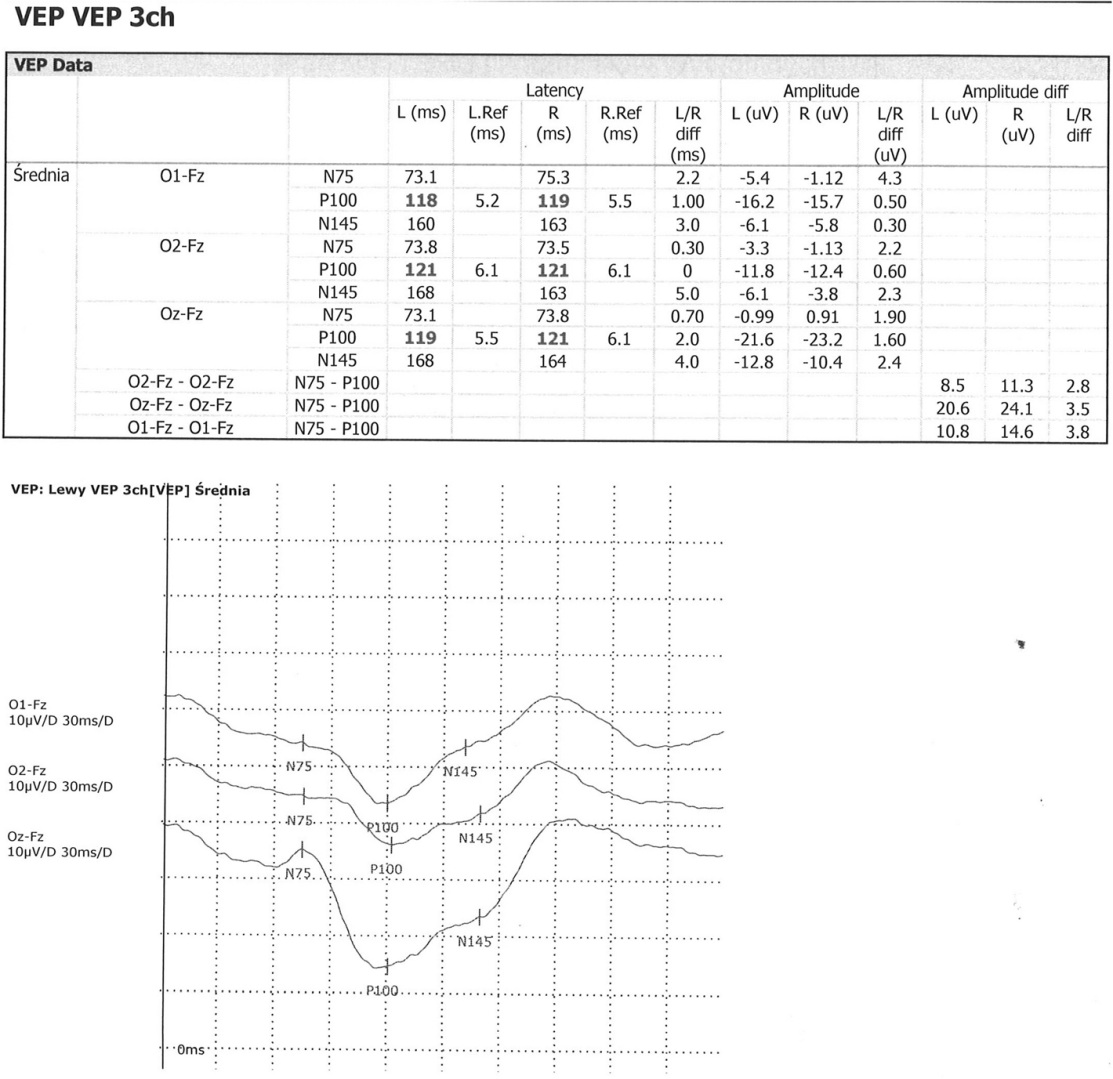
Abnormal VEP in 9 y.o. boy after ALL treatment

All patients were examined in comfortable position in shady room (about 50 lx). Before examination all patients have been evaluated for visual acuity. VEP were examined according to IFCN guidelines with using “pattern reversal” method. Black and white checkerboard pattern with specified dimension as 16 min of arc, was moving in sequence with frequency of 2 Hz. A monitor was 1 m in front of patient’s eyes. Average luminance of stimulator in middle of a stimulation area was 50 cd/m^2^. In study were used: Recording electrode placed in middle of occipital area, references electrodes in middle line on forehead in first scheme and two on auricles in the others and zero electrode on vertex. Used filter excluded frequencies below 0.3 Hz and above 300 Hz. Time of analysis was from 250 to 450 ms. At least 100 measurements were averaged. From obtained curve, positive (N70, N135) and negative (P100) were isolated for further analysis of their latencies and amplitudes. Only pairs of curves were evaluated in which the latency of the P100 wave did not differ by more than 2.5 ms (Figs. 3 and 4).

### Somatosensory evoked potentials methodology

**Fig. 5:**
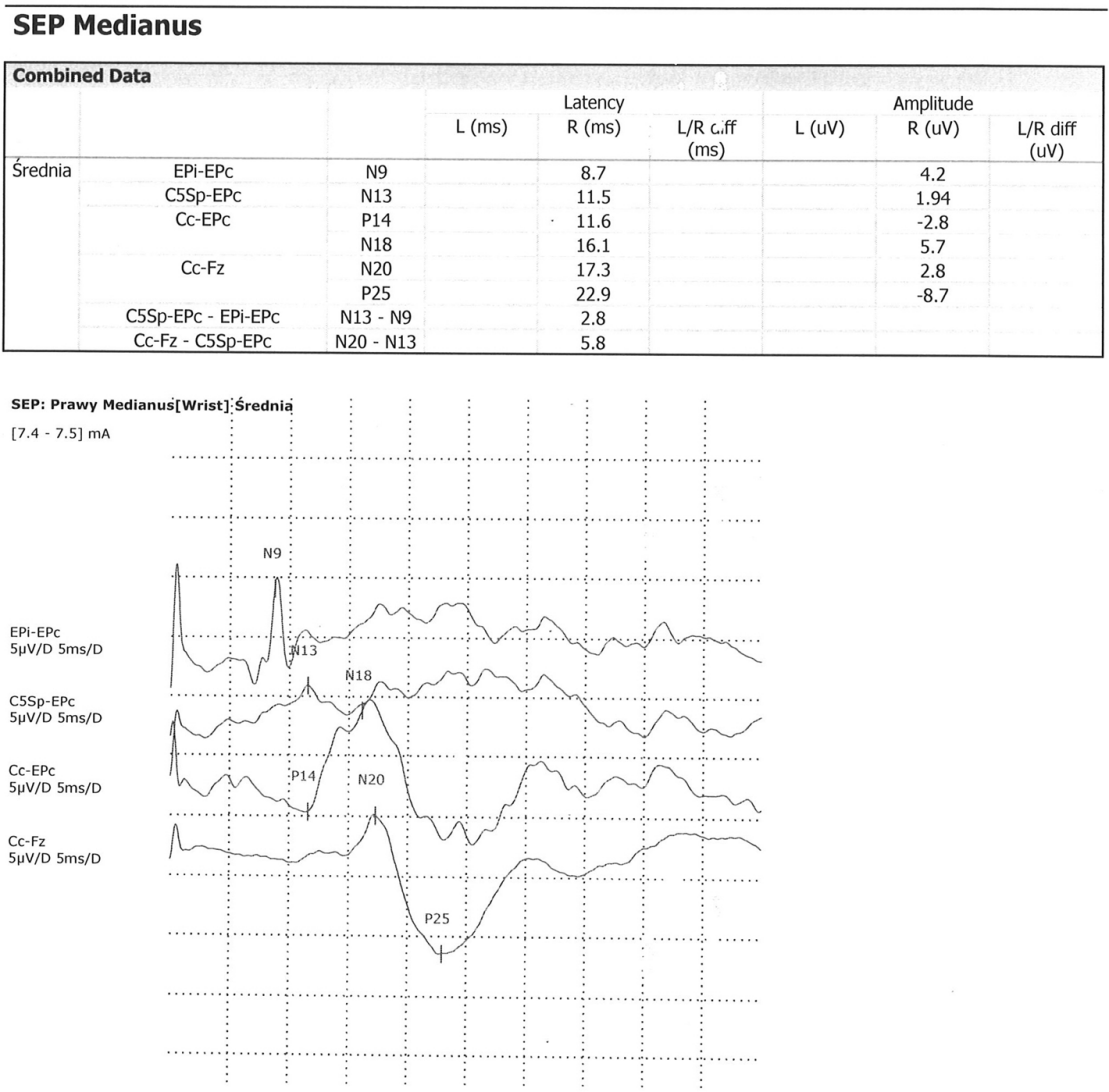
Normal SEP in 15 y.o. boy

**Fig. 6:**
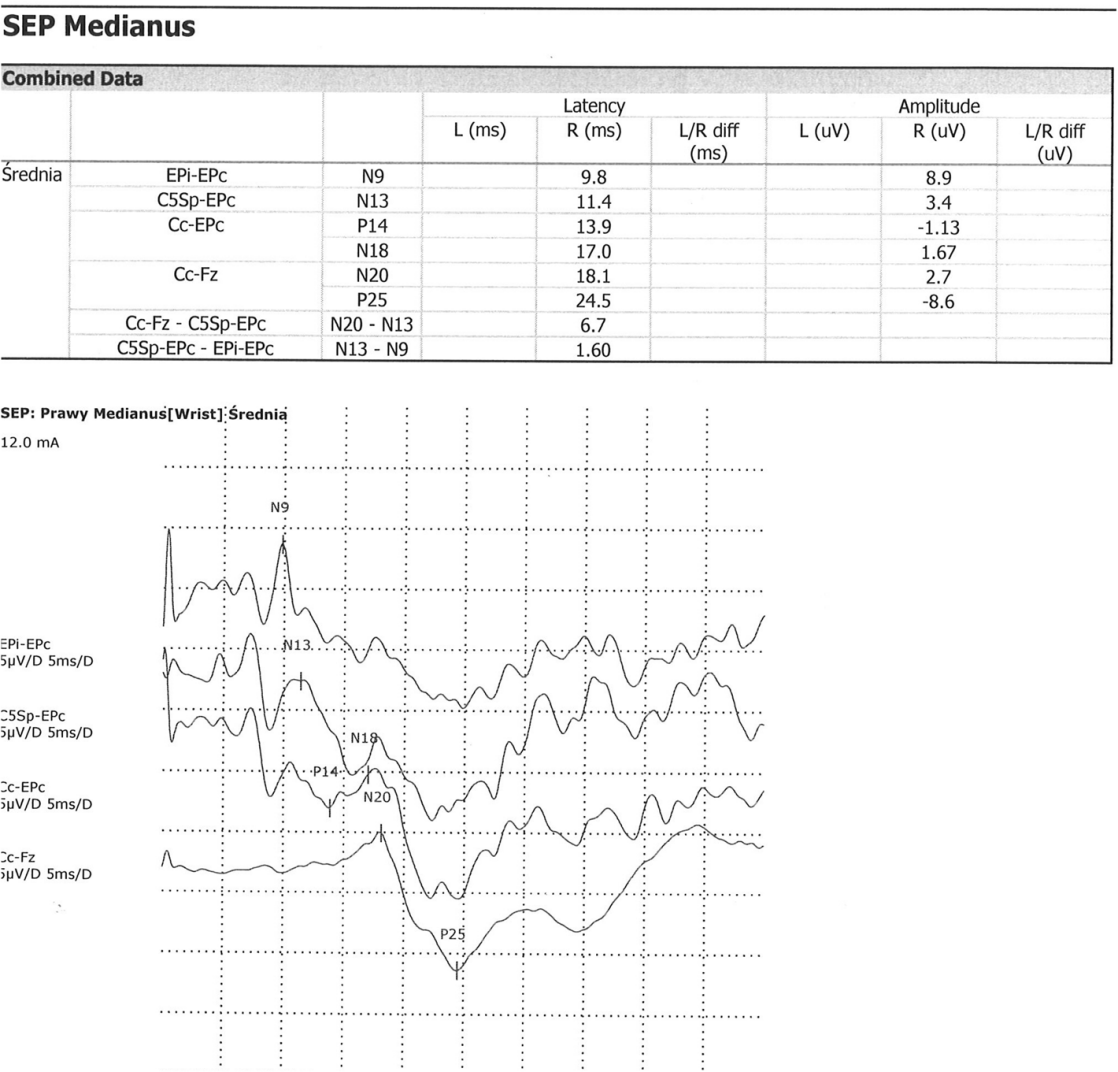
Abnormal SEP with prolonged N9 latency in 13 y.o. boy after ALL treatment

The SEP analysis was performed in accordance with IFCN recommendations. The median nerve was stimulated with repeated rectangular electric stimulus from electrode placed over the wrist area, with duration of 200 μs, frequency 5 Hz and intensity exceeding 3 times the threshold stimulus causing the sensory response. Response was recorded by 4 cup electrodes. In every case, before stimulation an electrode resistance was measured (lower than 500 Ω). Recorded frequencies below 20–30 Hz and above 3000 Hz were excluded. Average time of analysis was 50 ms. Depending on the legibility of the waves, 500–2000 responses were averaged. In every case 2 similar entries were recorded in which the latency differ not more than 0,25 ms and amplitudes no more than 20%. Amplitudes and latencies of above described waves were analyzed and compared to control group. Peripheral (PCT) and central (CCT) conduction times were estimated for differentiation. Results of examination was compared to our normal values based on own material. SEP waves with latency and interlatency elongation more than 2 SD and/or amplitudes less than 1 SD were assumed as abnormal (Figs. 5 and 6). P14 waves were not analyzed due to high variability in the control group.

### Statistical analysis

Statistical analyses were performed with Statistica 12.0 (StatSoft, Statistica 12.0, Tulsa, Oklahoma, USA) software. Continuous variables were expressed as mean ± standard deviation and categorical variables as number (percentage). Continuous variables were first checked for normal distribution by the Shapiro-Wilk statistic. Differences among two groups were compared by student’s t-test when normally distributed or by the Mann-Whitney test with test for non-normally distributed variables. In turn, differences among the three groups were compared by ANOVA test when normally distributed or by the Kruskal-Wallis test with test for multiple comparisons for non-normally distributed variables. Categorical variables were analyzed by the chi-square test and Fisher’s exact test depending on the size of the analyzed groups. *P*-value of less than 0.05 was considered statistically significant.

## Results

Significant age differences were observed in terms of treatment introduction and neurological control. Patients from pBFM group were significantly the youngest at the start of treatment (mean age 4.0 ± 2.7 years, *P* < 0.001). On the other hand, at the moment of neurological evaluation they were the oldest (18.3 ± 4.2 years, *P <* 0.001). Mean age of treatment introduction was 6.5 ± 0.5 years in NY and 5.0 ± 2.6 years in BFM95 and age of study - 14.0 ± 5.6 and 11.4 ± 4.1 years, respectively. Moreover, mean age of the control group was 11.6 ± 3.6 years and was significantly lower than age of ALL patients at study (13.9 ± 5.3 years, *P* = 0.016). However, we did not observe significant differences in term of gender distribution.

### Analysis of auditory evoked potentials in the study groups

Introduction of an oncological treatment contributed to observed shortening of latency of I wave (1.59 vs 1.90, *P* = 0.003) and to the prolongation of I-III interwave latency (2.23 vs 2.04, *P* = 0.004) and I-V (4.57 vs 4.24, *P* = 0.002) interwave latencies. Similar frequency of abnormalities in each protocol was observed (NY – 68.6%, pBFM – 61.7%, BFM95–69.4%). Moreover, there were no significant differences in frequency of wave and interwave latencies elongation between treatment groups (Table 3). Only average of interwave I-V latency in ALL survivor were significant longer than in control group (*P* = 0.035). Similar relationships were observed in additional comparison NY vs control group (*P =* 0.002) and BFM95 vs control group (*P* = 0.011). However, the latency of wave I was significantly longer in control group than in others.

**Table 3:** Auditory evoked potentials in the study groups

	NY	pBFM	BFM95	Control	*P* value
Prolonged latency of wave I	3 (8.6)	4 (8.5)	6 (7.1)	–	0.872
Prolonged latency of wave III	5 (14.3)	10 (21.3)	21 (24.7)	–	0.406
Prolonged latency of wave V	5 (14.3)	9 (19.2)	14 (16.5)	–	0.658
Prolonged interwave I-III latency	13 (37.1)	13 (27.7)	29 (34.1)	–	0.795
Prolonged interwave III-V latency	10 (28.6)	16 (34.0)	28 (32.9)	–	0.610
Prolonged interwave I-V latency	17 (48.6)	17 (36.2)	44 (51.8)	–	0.307
Latency of wave I	1.52	1.75	1.64	1.91	0.001*
Latency of wave III	3.82	3.96	3.87	3.98	0.615
Latency of wave V	6.14	6.27	6.14	6.16	0.990
Interwave I-III latency	2.21	2.19	2.26	2.04	0.081
Interwave III-V latency	2.33	2.33	2.34	2.20	0.562
Interwave I-V latency	4.58	4.52	4.59	4.24	0.035

* NY vs control *P* = 0.002, BFM95 vs control *P* = 0.011

Abbreviations: *BFM* Berlin-Frankfurt-Münster protocol, *NY* New York protocol

### Analysis of visual evoked potentials in the study groups

Due to technical difficulties, the examination was not performed in one patient from the BFM95 group. Direct comparison of the whole ALL survivors group to the control group revealed significant elongation of P100 (106.32 vs 101.57, *P* < 0.001) as well as N135 (151.42 vs 138.22, *P <* 0.001) latencies in the first one. Moreover, diffierences in total number of stated abnormalities were not observed (NY – 68.6%, pBFM – 42.5%, BFM95–58.3%). Analysis of examined VEP showed no significant differences between groups of patients after ALL treatment in occurrence of N70, P100 and N135 latency prolongation (Table 4). A significant difference was observed in a P100 amplitude decrease (*P* = 0.001). It was more often stated in the NY group (17.1%) than in others (pBFM - 2.1%, BFM - 0%). More distinctions were observed in comparison of average values to control group. Latencies of P100 (*P* = 0.005) and N135 (*P* < 0.001) were significantly longer in particular groups after ALL treatment. Noteworthy, P100 amplitude in BFM95 was significantly higher than in other treatment groups and control (*P* = 0.019).

**Table 4:** Visual evoked potentials in the study groups

	NY	pBFM	BFM95	Control	*P* value
Prolonged N70 latency	6 (17.1)	3 (6.4)	6 (12.2)	–	0.375
Prolonged P100 latency	13 (37.1)	10 (21.3)	31 (63.3)	–	0.541
Decreased P100 amplitude	6 (17.1)	1 (2.1)	0 (0.0)	–	0.001
Prolonged N135 latency	22 (62.9)	17 (36.2)	43 (87.8)	–	0.786
N70 latency	71.82	69.35	69.62	70.71	0.355
P100 latency	107.23	103.86	107.32	101.62	0.005*
P100 amplitude	10.17	9.24	12.55	12.31	0.019
N135 latency	152.17	148.18	153.03	138.22	< 0.001**

* NY vs control *P* = 0.050, BFM95 vs control *P* = 0.044

** NY vs control *P =* 0.001, pBFM vs control *P* = 0.028. BFM95 vs control *P* < 0.001

Abbreviations: *BFM* Berlin-Frankfurt-Münster protocol, *NY* New York protocol

### Analysis of somatosensory evoked potentials in the study groups

Due to technical difficulties, the examinations were not performed in two patients from the BFM95 group. Analysis of a total ALL group revealed that the anticancer treatment had a direct impact on amplitude reduction of N18 (3.24 vs 4.70, *P* = 0.007) and shortening of P25 latency (21.32 vs 23.39, *P* < 0.001). Moreover, there was also a significant difference in a total number of abnormalities between groups (NY – 62.9%, pBFM – 36.2%, BFM95–53.0%, *P* = 0.045). In collected data abnormal low amplitude was significantly most often in pBFM group for N13, N20, P25 and in BFM95 for N9. An occurrence of N18 amplitude decrease was comparable (*P* = 0.279). Abnormal prolonged latency was observed for N13 (*P* = 0.005) and N18 (*P* < 0.001) only in NY group. For other analyzed waves it was not observed. Next, average values of amplitude and latencies for each feature were compared to control group. N18 amplitude was significantly lower in ALL survivors groups than in control (*P* = 0.001). However, patients in control group had significantly longer P25 latency than groups after treatment (*P <* 0.001). Moreover suchlike relationship was observed in comparison of control to specific treatment group: NY vs control (*P <* 0.001), pBFM vs control (*P <* 0.001), BFM95 vs control (*P <* 0.001). Conduction times for PCT and CCT were not significantly often prolonged between ALL survivors groups and not significantly longer in comparison to the control group (Table 5).

**Table 5:** Somatosensory evoked potentials in the study groups

	NY	pBFM	BFM95	Control	*P* value
Prolonged N9 latency	0 (0.0)	0 (0.0)	0 (0.0)	–	–
Decreased N9 amplitude	0 (0.0)	10 (21.3)	18 (21.7)	–	< 0.001
Prolonged N13 latency	4 (11.4)	0 (0.0)	0 (0.0)	–	0.005
Decreased N13 amplitude	0 (0.0)	7 (14.9)	4 (4.8)	–	0.018
Prolonged P14 latency	3 (8.8)	2 (4.3)	4 (4.8)	–	0.930
Prolonged N18 latency	20 (57.1)	0 (0.0)	0 (0.0)	–	< 0.001
Decreased N18 amplitude	14 (40.0)	17 (36.2)	22 (26.5)	–	0.279
Prolonged N20 latency	2 (5.7)	0 (0.0)	2 (2.4)	–	0.250
Decreased N20 amplitude	0 (0.0)	7 (14.9)	4 (4.8)	–	0.018
Prolonged P25 latency	0 (0.0)	0 (0.0)	0 (0.0)	–	–
Decreased P25 amplitude	0 (0.0)	7 (14.9)	4 (4.8)	–	0.018
Prolonged PCT	3 (8.8)	1 (2.1)	8 (9.6)	–	0.270
Prolonged CCT	3 (8.8)	2 (4.3)	7 (8.4)	–	0.642
N9 latency	9.18	9.21	8.75	9.22	0.010
N9 amplitude	5.26	6.10	6.98	6.15	0.030
N13 latency	12.19	12.13	11.73	11.95	0.137
N13 amplitude	2.27	2.37	3.16	2.88	0.223
P14 latency	13.67	13.80	13.39	13.17	0.170
P14 amplitude	1.80	2.17	1.73	2.12	0.695
N18 latency	16.69	16.94	16.50	17.17	0.117
N18 amplitude	3.12	2.53	3.70	4.70	0.001*
N20 latency	18.39	18.21	17.75	17.95	0.070
N20 amplitude	1.86	1.66	1.72	2.24	0.356
P25 latency	21.65	21.25	21.22	23.39	< 0.001**
P25 amplitude	3.88	3.58	3.90	4.43	0.600
PCT	3.01	2.91	2.97	2.73	0.467
CCT	6.20	6.09	6.03	6.00	0.882

* NY vs control *P =* 0.042, pBFM vs control *P =* 0.002

** NY vs control *P <* 0.001, pBFM vs control *P <* 0.001, BFM95 vs control *P <* 0.001

Abbreviations: *BFM* Berlin-Frankfurt-Münster protocol, *CCT* central conduction time, *NY* New York protocol, *PCT* peripheral conduction time

### Impact of radiotherapy on evoked potentials

To evaluate the effect of radiotherapy on EP, additional analysis was performed in above-mentioned alternative groups (NY + pBFM vs BFM95). There were no significant differences in the summary recorded features of particular EP (BAEP: 64.6 vs 69.4%, VEP: 53.7 vs 58.4%, SEP: 47.6 vs 53.0%). Moreover, significant differences were also not observed in BAEP parameters between compared groups (Table 6). However, the conducted analysis of VEP has shown that in NY + pBFM group, amplitude of P100 was significantly more often decreased (8.5 vs 0%, *P* = 0.006) and P100 amplitude mean value was significantly lower (9.61 vs 12.46, *P* = 0.004) (Table 7). Other analyzed features of VEP were comparable between groups. Finally, the effect of radiotherapy on SEP was investigated (Table 8). Patients treated with radiotherapy significantly more frequently represented a prolonged N18 latency (24.4 vs 0%, *P* < 0.001). Also the mean value of N18 amplitude was significantly lower in NY + pBFM group (*P* = 0.009). However, direct comparison has shown that in group BFM95 (21.7%) amplitude of N9 was (*P* = 0.042) decreased more often than in joint group with radiotherapy (12.2%). Further analyses shown that in NY + pBFM group latencies were significantly longer in: N9 (*P* = 0.002), N13 (*P* = 0.018), N20 (*P* = 0.012). It corresponds with significant reduction of amplitude for: N9 (*P* = 0.007), N13 (*P* = 0.038), N18 (*P* = 0.009).

**Table 6:** Auditory evoked potentials in irradiated and non-irradiated groups

	NY + pBFM	BFM95	*P* value
Prolonged latency of wave I	7 (8.5)	6 (7.1)	0.722
Prolonged latency of wave III	15 (18.3)	21 (24.7)	0.314
Prolonged latency of wave V	14 (17.1)	14 (16.5)	0.917
Prolonged interwave I-III latency	26 (31.7)	29 (34.1)	0.740
Prolonged interwave III-V latency	26 (31.7)	28 (32.9)	0.865
Prolonged interwave I-V latency	34 (41.5)	44 (51.8)	0.182
Latency of wave I	1.61	1.57	0.621
Latency of wave III	3.82	3.84	0.776
Latency of wave V	6.15	6.14	0.909
Interwave I-III latency	2.20	2.26	0.367
Interwave III-V latency	2.33	2.34	0.889
Interwave I-V latency	4.55	4.59	0.646

Abbreviations: *BFM* Berlin-Frankfurt-Münster protocol, *NY* New York protocol

**Table 7:** Visual evoked potentials in irradiated and non-irradiated groups

	NY + pBFM	BFM95	*P* value
Prolonged N70 latency	9 (11.0)	6 (12.2)	0.389
Prolonged P100 latency	23 (28.1)	31 (63.3)	0.223
Decreased P100 amplitude	7 (8.5)	0 (0.0)	0.006
Prolonged N135 latency	39 (47.6)	43 (87.8)	0.640
N70 latency	70.37	69.58	0.485
P100 latency	105.27	107.34	0.158
P100 amplitude	9.61	12.46	0.004
N135 latency	149.78	153.01	0.171

Abbreviations: *BFM* Berlin-Frankfurt-Münster protocol, *NY* New York protocol

**Table 8:** Somatosensory evoked potentials in irradiated and non-irradiated groups

	NY + pBFM	BFM95	*P* value
Prolonged N9 latency	0 (0.0)	0 (0.0)	–
Decreased N9 amplitude	10 (12.2)	18 (21.7)	0.042
Prolonged N13 latency	4 (4.9)	0 (0.0)	0.339
Decreased N13 amplitude	7 (8.5)	4 (4.8)	0.339
Prolonged P14 latency	5 (6.1)	4 (4.8)	0.717
Prolonged N18 latency	20 (24.4)	0 (0.0)	< 0.001
Decreased N18 amplitude	31 (37.8)	22 (26.5)	0.120
Prolonged N20 latency	2 (2.4)	2 (2.4)	0.989
Decreased N20 amplitude	7 (8.5)	4 (4.8)	0.339
Prolonged P25 latency	0 (0.0)	0 (0.0)	–
Decreased P25 amplitude	7 (8.5)	4 (4.8)	0.339
Prolonged PCT	4 (4.9)	8 (9.6)	0.239
Prolonged CCT	5 (6.1)	7 (8.4)	0.563
N9 latency	9.20	8.75	0.002
N9 amplitude	5.74	6.98	0.007
N13 latency	12.15	11.73	0.018
N13 amplitude	2.32	3.16	0.038
P14 latency	13.74	13.39	0.100
P14 amplitude	2.01	1.73	0.429
N18 latency	16.83	16.50	0.139
N18 amplitude	2.78	3.70	0.009
N20 latency	18.29	17.75	0.012
N20 amplitude	1.74	1.72	0.924
P25 latency	21.42	21.22	0.460
P25 amplitude	3.71	3.90	0.639
PCT	2.95	2.97	0.857
CCT	6.14	6.03	0.564

Abbreviations: *BFM* Berlin-Frankfurt-Münster protocol, *CCT* central conduction time, *NY* New York protocol, *PCT* peripheral conduction time

## Discussion

The aim of our study was to present the possibilities of complex EP analysis application in a large group of 167 ALL long-term survivors. As we have shown, the oncological treatment had a significant impact on the shape of registered EP. The disturbances, mainly in the form of longer conduction time, were recorded in a significant percentage of patients in each of the analyzed protocols. As we have already emphasized above, the impact of radiotherapy used in NY and pBFM protocols was most clearly marked in SEP analysis. According to our knowledge, our study is the largest study dedicated to the issue of EP in ALL survivors so far. Also, the analyzed follow-up time was one of the longest in literature. Moreover, our study is only the third report on the simultaneous analysis of several EP types in one homogenous ALL study group.

Published EP studies have not consolidated their final position in the diagnosis of ALL survivors. As we mentioned earlier, only few studies presenting complex assessment with different types of EP can be found in the literature [9, 11, 12]. Kaleita et al. [9] found abnormalities in individual EP in a significant percentage of patients (50% - VEP, 41.7% - BAEP, 36.4% - SEP). However, their analyses were based on both patients with ALL as well as with acute myeloblastic leukemia qualified for bone marrow transplantation. Muchi et al. [11] conducted their study on a homogeneous group of ALL patients who survived at least 2 years since the end of oncological treatment. The small group of 28 participants, however, makes it impossible to draw final conclusions and this report should be interpreted as a preliminary observation. The study by Korinthenberg and Igel [12] on 79 ALL patients who completed anticancer treatment at least 18 months earlier has been the most comprehensive report to date. As observed, changes in VEP and BAEP were transient and regressed after the observation period. Moreover, no significant differences were found between irradiated and non-irradiated patients. The most important report for VEP was the study by Russo et al. [10]. The delay in conductivity in VEP analysis in almost all asymptomatic patients who received radiotherapy created the basis for further research on this phenomenon. Much fewer reports in the literature concerned the use of BAEP. One of them is our report from 2006, which showed the presence of abnormalities in 22.4% of asymptomatic ALL patients [8]. Moreover, the role of SEP in ALL patients has been extensively studied in Finnish centers [15–17]. The neurotoxic effects of vincristine and intrathecal methotrexate have been demonstrated several times in various parts of the nervous system.

According to the literature BAEP is highly sensitive in detection of nerve VIII tumors, abnormalities were noticed in above 90% of patients with acoustic neurinoma. BAEP abnormalities observed in acoustic neurinoma and cerebellopontine angle tumors oscillate from elongation of interlatencies of I-III waves (what indicates conduction delay between distal part of VIII nerve and lower part of the pontine) to disappearance of the components of the response III and V, or total disappearance of all components. Other changes of SPWPM parameters were observed in patients with neoplastic diseases and without structure brain stem damage [12, 18–20]. In patients with ALL in treatment subgroups all interlatencies were elongated. Although statistically significant differences between groups were noticed only in interlatencies I-V (in ANOVA and Kruskal-Wallis tests), in other tests the significant differences were not observed in comparison between studied subgroups and subgroups and control. Clear evidence in ALL group showing slowing of the conductivity of auditory stimuli in CNS, was significant elongation of interlatencies I-III and I-V in comparison to control. Another interesting observation was elongation of BAEP latency and interlatency observed slightly more often in patients treated according to BFM 95 protocol (69,41%), what can be explained by shorter time interval between completion of therapy and testing. Of importance seems to be the observation that was no statistically significant influence of radiotherapy on BAEP parameters. The obtained data should be treated with caution. High prevalence of patients with detected elongation of BAEP parameters did not translate into significant increase of mean latency BAEP parameters. In the context of our results it seems that measurements of I-V interlatencies are the best markers in neurophysiological assessment of hearing impairment in patients treated because of ALL.

Uberall et al. [14] shown that frequency and severity VEP changes in patients with and without radiotherapy was similar. This is in a discrepancy with our results indicating significant unfavorable influence of radiotherapy on P100 potential amplitude value.

In our study abnormalities of VEP parameters were shown in 93 (56%) of patients treated due to ALL and the most often with NY protocols. Radiotherapy had not significantly important adverse influence on lowering the amplitude of the P100 wave, however, it had no also adverse effect on elongation of its latency. An interesting observation seems to be the fact that N135 latency was significantly longer between ALL subgroups than in comparison between all studied ALL subgroups and control group. In turn, of statistical analysis of SEP did not confirmed preliminary observations suggesting significant elongation of central conduction time (CCT) published previously, which probably resulted from the smaller size of the group assessed at that time. Ultimately, however, a slight prolongation of PCT and CCT depending on the intensity of the therapeutic program was demonstrated in comparison of all studied subgroups and control. A similar relationship could be seen in most mean SEP between patients treated with radiotherapy and BFM95 (without radiotherapy). Our data is in accordance with observed by Vainionpää et al. [15], who studied SEP in 38 children with ALL, elongations of PCT time were observed in the group of children with standard, medium and high risk.

It can be stated that the most frequently used argument in studies negating the usefulness of the EP analysis in ALL patients is the lack of their translation into the abnormalities found in the physical examination [13, 14]. This lack of correlation can be found especially in the case of VEP and BAEP analyses. Also in the current study, abnormalities in BAEP, VEP and SEP were found in a large percentage of asymptomatic patients. Several potential explanations for this disproportion have been proposed so far in the literature. Newman et al. explained from the ophthalmologic side that excellent visual acuity is usually retained despite marked neuroocular dysfunction [21]. Therefore, there is a wide margin for subclinical damage of the visual pathway. Subclinical abnormalities in BAEP can be explained by the subclinical functional defect of cochlea induced by a radiation [12].

In our study, we analyzed ALL long-term survivors, aged 4.9–28.4 years. Therefore, all our patients were at the age at which common comorbidities such as diabetes and hypertension, which may contribute to further loss of nerve fibers, are not common [21]. Furthermore, as has been shown many times, long-term ALL survivors are a high-risk group for developing diabetes [22] as well as hypertension [23]. For this reason, we hypothesized that the one-time screening with EP presented by us will allow to separate a group of patients at increased risk of developing clinically apparent visual, hearing or sensory impairment. Currently, there is no research in the literature that could confirm this initial hypothesis. For this reason, longer observations also involving genetic methods useful in the pediatric population [24, 25] are necessary.

Neurophysiological assessment allows to make evaluation of patient health objective. As EP is noninvasive method, it can be repeatedly performed, being good indicator of recovery or progression. It seems to be the best tool to neurologic late effects monitoring. EP allows to detect changes even many years after completing of therapy. Together with precise clinical evaluation EP allows to correct the conclusions from individual evaluation of students and their professional activation opportunities after ALL therapy. High prevalence of detected abnormalities in the neurophysiological assessments indicates necessity of its monitoring in prolonged follow-up after treatment. Although observed abnormalities are not severe, they can explain lower psychophysical capacity of youth entering adult life, being treated for years with multimodal chemotherapy/radiotherapy during childhood. Questionnaires used in many study protocols, evaluating quality of life are very useful in the assessment. Unfortunately subtle peripheral and central nervous system dysfunctions may not be detected, and patients can be unjustly treated by society as persons psychophysically healthy. Therefore precise clinical evaluation should be in accordance with indications replenished by other neurophysiological technics, including evoked potentials.

Our study has several limitations. First, the analyzed patient groups differed in terms of age. However, it has been shown that age is not a significant factor modulating EP [26]. Second, in our study we analyzed asymptomatic patients. Therefore, we could not compare the results of the EP analysis with the abnormalities in the neurological examination. Third, in the current study we analyzed ALL protocols used in clinical practice in the past. However, only such an analysis made it possible to verify the impact of radiotherapy on EP, which was one of the objectives of our studies [27].

## Conclusions

The oncological treatment had a significant impact on the shape of registered EP. The presented analysis indicates a high frequency of subclinical abnormalities in EP regardless of the analyzed protocol. To our knowledge current study is the largest and one of the most complex research with the longest follow-up time examining the role of EP in ALL patients. The obtained results indicate the possibility of using a single, objective and non-invasive measurement of EP in ALL survivors in order to stratify the risk of developing sensory abnormalities in adulthood.

## Data Availability

The datasets used and analysed during the current study are available from the corresponding author on reasonable request.

## Abbreviations

ALL: Acute lymphoblastic leukemia
EP: Evoked potentials
NY: New York protocol
BFM: Berlin-Frankfurt-Münster protocol
VEP: Visual evoked potentials
SEP: Somatosensory evoked potentials
BAEP: Brainstem auditory evoked potentials
IFCN: International Federation of Clinical Neurophysiology
PCT: Peripheral conduction time
CCT: Central conduction time
